# Influence of Plant Secondary Metabolites on intake, Detoxification Costs, and Microbial Communities in Deer

**DOI:** 10.1007/s10886-026-01729-z

**Published:** 2026-06-20

**Authors:** Katie L. Anderson, Lisa A. Shipley, Anna R. Staudenmaier, Stephanie J. Galla, Jennifer S. Forbey

**Affiliations:** 1 School of the Environment, Washington State University, Pullman, WA USA; 2 Research Corporation of the University of Hawai’i Hilo, Hilo, HI USA; 3 Department of Biological Sciences, Boise State University, Boise, ID USA

**Keywords:** Detoxification limitation hypothesis, Glucuronic acid, Microbiome, Monoterpenes, *Odocoileus*, Phenolics

## Abstract

Plants available to wild herbivores, especially browsers, often contain plant secondary metabolites (PSMs). Herbivores have evolved behavioral, physiological, and microbial mechanisms for avoiding and detoxifying PSMs. The detoxification limitation hypothesis suggests that herbivores can reduce toxicity by consuming a mixture of PSMs to avoid overloading a particular detoxification pathway. Although this hypothesis has been examined for smaller mammalian hindgut-fermenters, less is known about responses to PSM mixtures in wild ruminants. To assess the role of host and microbial responses to PSM composition, we used controlled feeding trials to measure voluntary dry matter and PSM intake, urinary excretion of glucuronic acid (GA, a byproduct of PSM detoxification through conjugation), and the diversity and relative abundance of gastrointestinal bacterial families in the feces of two species of captive-raised deer (*Odocoileus hemionus*,* O. virginianus*). Deer were fed five mixtures of four purified PSMs that included two same-chemical class mixtures, two different-class mixtures, and one 4-way mixture of all chemicals. Overall, we found that PSM composition had minimal effect on intake, that GA was a consistent physiological biomarker of PSM intake regardless of PSM composition, and that dietary phenolics may influence microbial communities more than monoterpenes. Our results *partially* conformed to the detoxification limitation hypothesis, where deer consumed less of one same-class mixture (monoterpenes) than different-class mixtures. Our results point to the complexity of the interplay between different behavioral, physiological, and microbial mechanisms that can mediate the consequences of PSMs.

## Introduction

To survive and reproduce, animals spend most of their lives eating or avoiding being eaten. For example, as large ruminant herbivores, moose (*Alces alces*) spend up to 75% of their time across seasons foraging and ruminating (Risenhoover 1986; Van Ballenberghe And Miquelle 1990; Dungan et al. 2010; Felton et al. 2016), but forage less and spend more time alert with increasing wolf predation risk (Steinhauer 2024). In contrast, smaller species or younger animals may need to spend more time avoiding predators and less time eating. Neonatal mule deer lie nearly motionless for more than 80% of their day, relying on cryptic coloration and lack of scent glands to avoid predation, and spending most of their remaining time nursing (Carl and Robbins 1988). Plants also face life history tradeoffs in growth, competition, and avoiding herbivory (Coley et al. 1985; Herms And Mattson 1992), such as maximizing sunlight for photosynthesis in forest openings verses maximizing crypticity that protects them from herbivory under the forest canopy (Glynn et al. 2007; Smilanich et al. 2016). As a consequence, adaptations and counteradaptations of food and forager often result in what has been classically characterized as a coevolutionary arms race (i.e., reciprocal evolution, Ehrlich And Raven 1964; Janz 2011).

In the face of herbivory, plants have developed several strategies for defending their tissues and reproductive products. In addition to mechanical defenses that include fibrous cell walls (Van Soest 1994; Shipley et al. 1999; Salgado-Flores et al. 2016) and thorns (Melewski et al. 1991; Medina-Villar et al. 2022), plants produce thousands of different chemicals (plant secondary metabolites, PSMs), many of which defend them against herbivores (Bryant et al. 1983; Hartmann 2007). PSMs are ubiquitous and especially common in woody and herbaceous dicots (Bryant et al. 1991, 1992; McArthur et al. 1993; Smilanich et al. 2016). They are classified by their chemical structure (e.g., phenolics, terpenoids, and alkaloids) and their effects on herbivores depend on their chemical properties that influence how fast they are absorbed and distributed in the body and pathways for detoxification and elimination (i.e., Forbey et al. 2013). Their effects on herbivores range from seemingly benign, to reducing energy or nutrient assimilation, to toxicity that damages tissues or causes death (Marsh et al. 2006a).

In turn, vertebrate herbivores employ behavioral, physiological, and microbial tactics to counteract the effects of PSMs (Iason And Villalba 2006; Marsh et al. 2006a; Kohl et al. 2016). Herbivores that choose to eat plants with PSMs can modulate how much they eat in a meal or day to maintain safe levels in their body (i.e., satiety hypothesisSorensen et al. 2005a; McLean et al. 2008; Torregrossa and Dearing 2009; Moore et al. 2015). However, reducing intake to avoid PSMs also reduces energy and nutrient intake, which might reduce fitness (DeGabriel et al. 2009a). Herbivores can also consume a mixture of plants with different types of PSMs to reduce overloading of any one of their detoxification pathways, also known as the detoxification limitation hypothesis (Freeland And Janzen 1974; Dearing et al. 2000; Marsh et al. 2006a).

If PSMs are ingested, some herbivores have physiological mechanisms to reduce their absorption (Green et al. 2004; Foley and Moore 2005; McLean And Duncan 2006; Sorensen and Dearing 2006). PSMs that are absorbed can be detoxified into water-soluble compounds in the liver and then excreted in the urine or bile. Phase I enzymes (cytochrome P450s [CYP]) do this through rate-limited oxidation, reduction, or hydrolysis reactions (Caldwell 1986; McArthur et al. 1991; Purdy et al. 2004; Guengerich 2002) that can be inhibited by PSMs (Pass and McLean 2002; Kimura et al. 2010). Phase II enzymes (transferases) conjugate the PSM to an endogenous polar molecule (e.g., glucuronic acid, glycine, Caldwell 1986; McArthur et al. 1991) and are primarily limited by the availability of energy dependent cofactors used in conjugation reactions (Thurman And Kauffman 1980; Aw and Jones 1982; Reinke et al. 1994). For example, when PSMs are detoxified via the glucuronidation pathway, the relative amount of GA excreted can be used as an index of total PSM intake and energetic cost via the loss of endogenous glucose comprising the glucuronide (Mangione et al. 2001; Dearing et al. 2005; Sorensen et al. 2005a, b; Servello And Schneider 2000; Parikh et al. 2017).

Herbivores can also use symbiotic microbes in their gastrointestinal (GI) tracts that can detoxify PSMs (Dearing And Weinstein 2022; Rogowska-van Der Molen et al. 2023). Because of short generation times, their community composition and adaptations of microbes can change relatively rapidly as food sources change (Arber 1991; Hammer And Bowers 2015; Bahrndorff et al. 2016). For example, the entire life cycle of an active strain of *E. coli* can occur within 20 min (Arber 1991). The various microbes provide a range of biological functions that benefit the herbivores (McBee 1971; Troyer 1984) and functional redundancy across the microbial community is thought to increase resiliency of the host-microbe system (Allison And Martiny 2008; Solden et al. 2018; Söllinger et al. 2018). Foregut fermenters such as ruminants may receive particular benefits from their GI microbes because they are primarily housed before the gastric stomach and the absorption sites in the small intestine (Van Soest 1994). Specifically, PSMs can be bio-transformed by microbes in the GI before entering the host’s detoxification system (Russell 2002).

**Fig. 1:**
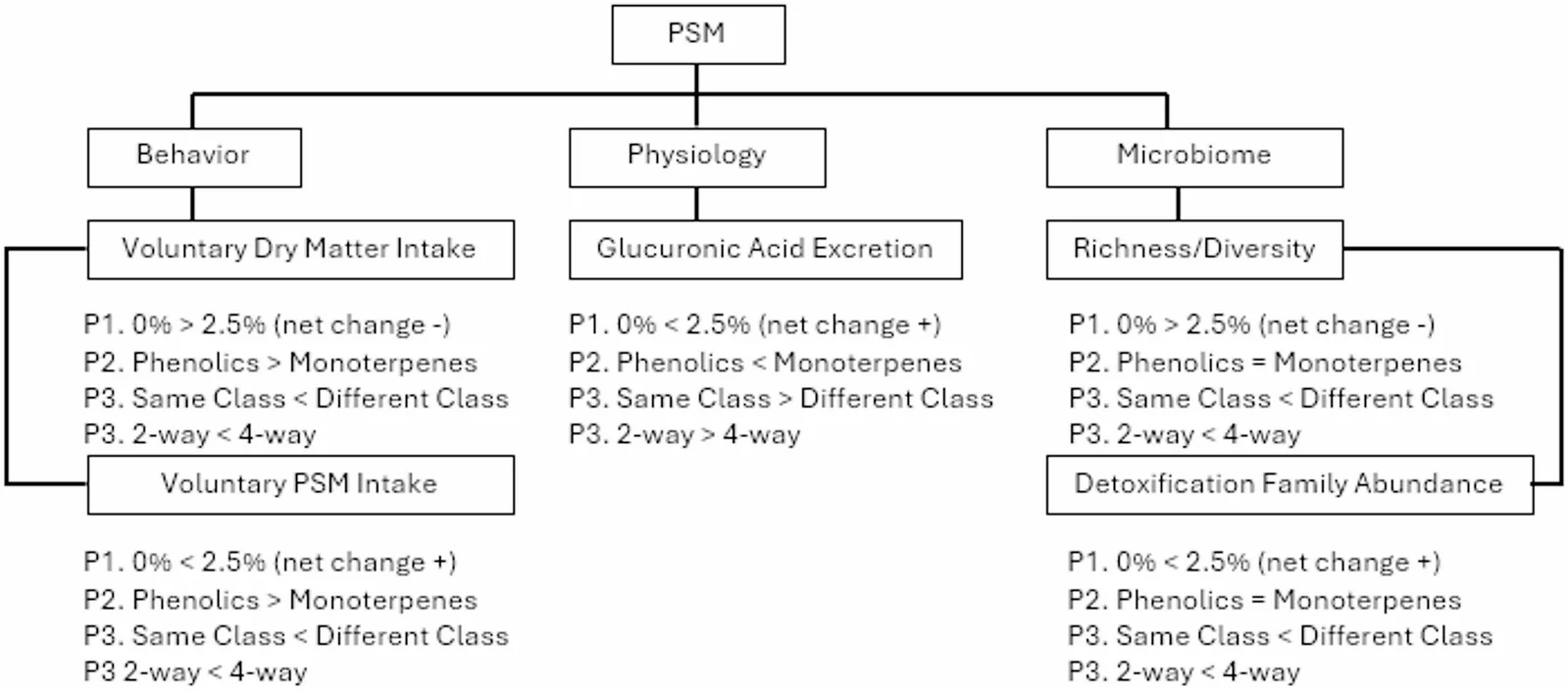
Predictions for how deer (*Odocoileus* spp.) will respond to 2.5% concentrations of dietary plant secondary metabolites (PSMs) compared to PSM-free diets (0%) when fed as mixtures of compounds in the same chemical class (either 2 monoterpenes or 2 phenolics) and different chemical classes (2-way: 1 monoterpene and 1 phenolic, 4-way: 2 monoterpenes and 2 phenolics. Behavioral responses included voluntary dry matter intake [g/day] and PSM intake [g PSM/day], physiological responses included excretion of glucuronic acid [mM GA/g PSM/day], and microbial responses included diversity and community composition of gastrointestinal microbial communities

Despite the importance of PSMs to the ecology of wild ruminants (Robbins et al. 1987; Spalinger et al. 2010; Berry et al. 2019) and the economic value of wild and domestic ruminants (Malecky et al. 2009a, b; Arnett And Southwick 2015; Heroy et al. 2018), little is known about how browsing ruminants respond simultaneously in terms of behavior, physiology, and microbial communities to varying composition of PSMs. Previous work has characterized behavioral, physiological, and microbial responses to dietary PSMs by small hindgut fermenters, especially woodrats (*Neotoma* spp., Sorensen et al. 2005a, b; Haley et al. 2007; Kohl and Dearing 2016; Schramm et al. 2024) and common brushtail possums *(Trichosurus vulpecula, *Pass and Foley 2000; Marsh et al. 2006b; McLean et al. 2008; DeGabriel et al. 2009b), but less is known about mechanistic responses in wild ruminants (but see also Sundset et al. 2007, 2010; Glad et al. 2014; Staudenmaier et al. 2022). To assess the role of host and microbial responses to PSM composition, we conducted a series of feeding trials with mule deer (*Odocoileus hemionus*) and white-tailed deer (*Odocoileus virginianus*) in which deer were fed mixture combinations of four PSMs (two phenolics and two monoterpenes) prevalent in forages they consume in their natural habitats in North America. We measured their responses in terms of behavior (daily dry matter intake [DMI, g/day] and daily PSM intake [PSMI, g/day]), physiology (production of GA [mM GA/g PSM/day]), and GI microbes (community richness [number of taxa], community diversity [Shannon diversity index], relative abundance of taxa). We tested three predictions based on the physiochemical traits of our specific PSMs and results from previous pharmacokinetic and pharmacological studies in other animals including specialist and generalists vertebrate herbivores (Table 1; Fig. 1).

**Table 1:** Physical and molecular characteristics of the chosen plant secondary metabolites chosen for controlled feeding trials including the plant secondary metabolites (PSM) added to a basal diet, mixed to equal 2.5% of the diet by mass, and given to captive mule deer (*Odocoileus hemionus*) and white-tailed deer (*Odocoileus virginianus*)0.3

Characteristic	Plant secondary metabolite			
	Salicin	Quercetin	1,8 cineole	α-pinene
Chemical class	Phenolic	Phenolic	Monoterpene	Monoterpene
Type of PSM	Phenolic glycoside	Flavonone	Bicyclic ether	Bicyclic monounsaturated
Molecular weight	286.26	302.24	154.25	136.23
Consensus logP (lipophilicity)	−0.78	1.23	2.67	3.44
CYP inhibition	CYP1A2 (Gomes et al. 2021)	CYP1A2, CYP2D6, CYP3A4 (Hai et al. 2020)	CYP2C9 (Pass and McLean 2002)	CYP2C9, CYP2DP (Deodhar et al. 2020; Green et al. 2004, 2006; Elmeliegy et al. 2020; Agreles et al. 2021; Dias et al. 2022)
CYP induction		CYP3A4 (Patel 2004, Yu 2011)		
Phase I pathways (microbes can be involved)	Primarily hydrolysis, secondarily oxidation (Fötsch et al. 1989; McLean et al. 2001)	Limited (Gaspar et al. 1994; Berger et al. 2012)	Oxidation (Miyazawa et al. 1989; Boyle et al. 1999,2000; Shipley et al. 2012)	Oxidation and hydrolysis ( Ishidax et al. 1981; Pass et al. 1999; Lamb et al. 2004; Ishida 2005; Schramm et al. 2024)
Known Phase I transformation products (host and/or microbes)	Salicyl alcohol Salicylic acid	Quercetin glycosides	Hydroxycineoles Cineolic acids Dihydroxycineoles Hydroxycineolic acids	Trans-verbenols Myrtenols Pinene-oxides Myrtenic acids Dihydromyrtenic acid Pinanols
Phase II	Major GA conjugation, minor glycine conjugation (Fötsch et al. 1989; McLean et al. 2001)	Presystemic methylation, sulfation, and GA conjugation (Spencer et al. 2003; Duenas et al. 2010; Berger et al.^2012^, ^2015^; Yeh et al. 2016; Hai et al. 2020)	Minor GA conjugation primarily after Phase I (Boyle et al. 2000; Shipley et al. 2012)	GA conjugation likely, but not specifically linked to α-pinene (Sorensen et al.^ 2005^; Schramm et al. 2024) except in humans (Xie et al. 2025)
Known Phase II transformation products (host and/or microbes)	Salicyl alcohol glucuronide Salicylic acid glucuronide Salicyluric acid (a glycine conjugate)	Methylquercetin–glucuronide Quercetin-3’-sulfate 4-methylcatechol 1-sulfate Isorhamentin-glucuronide sulfate	Hydroxycineole glucuronide, Cineolic acid glucuronide, Dihydroxycineole glucuronide, Hydroxycineolic acid glucuronide	Myrtenic acid glucuronide Dihydromyrtenic glucuronide

Our first prediction (P1) was that when fed diets containing purified PSMs at relatively low concentrations, similar to the concentrations found in natural diets, deer would consume less dry mass of food but more mass of PSMs and excrete more GA than when consuming a PSM-free diet. This relationship was shown in previous studies of deer consuming single PSM diets (Staudenmaier et al. 2022) and natural browses (Servello And Schneider 2000). In addition, we predicted that when deer consumed more PSMs they would have a lower overall diversity of GI microbial taxa because of potential antimicrobial effects (Berger et al. 2015; Cosme et al. 2020; Wink 2022) but a higher relative abundance of microbial taxa associated with degrading PSMs (Kohl et al. 2016).

Second, we predicted higher intake and lower GA when deer consume diets with phenolics than monoterpenes fed at the same concentration (P2, Fig. 1). Although detoxification pathways of PSMs have yet to be investigated in deer, our prediction was based on the molecular structure of these two chemical classes (Table 1). Phenolics are hydrophilic (more polar), whereas monoterpenes are lipophilic, thus phenolics have a much lower, and often negative, LogP scores (lipophilicity) than monoterpenes. As such, hydrophilic phenolics and are expected to have longer retention in the rumen and the lower GI tract where they would interact more with GI microbes (Griffiths et al. 1987; Trudgill 1990) but have lower absorption resulting in lower systemic exposure and need for glucuronidation in the host animal compared to monoterpenes. Our alternative prediction is that intake of phenolics would result in higher GA excretion than intake of monoterpenes because phenolics are generally more reliant on glucuronidation before and after absorption (Gou And Bruno 2015), whereas monoterpenes rely on oxidation or hydrolysis using CYPs (Phase I) often followed by glucuronidation (Phase II, Table 1, Gou And Burno 2015; Shipley et al. 2012).

Third, based on the detoxification limitation hypothesis (Freeland And Janzen 1974), we predicted that deer would consume less dry mass and PSM mass on diets that contain individual PSMs from the same chemical class (i.e., two phenolics or two monoterpenes) than when consuming mixtures of different chemical classes (i.e., monoterpenes and phenolics, P3, Fig. 1; Table 1). The detoxification limitation hypothesis has found some support from controlled experiments with wild herbivores (e.g., Dearing et al. 2000; Marsh et al. 2006b; Rogosic et al. 2007; McLean et al. 2008) and posits that herbivores and presumably their GI microbial communities possess numerous detoxification enzymes that can be saturated by the intake of PSMs that are primarily detoxified through the same enzymes. Spreading the detoxification burden more equally or over more pathways when consuming mixtures of PSMs from different chemical classes is also expected to reduce GA production per unit of PSM consumed than when consuming the same classes. Alternatively, given that both parent PSMs and those metabolized by Phase I enzymes can be glucuronidated and assuming deer are not energy limited, GA production may be dependent on the intake of PSM regardless of chemical class. Overall, we expected microbial community richness and diversity to be higher when the deer consumed a greater diversity of PSMs because mixtures would promote distinct microbes that can use or bio-transform distinct chemicals (Marsh et al. 2006b; Richards et al. 2016; Jing et al. 2020).

## Methods and Materials

### Selection of PSMs

We tested our predictions using four PSMs, two phenolics (salicin and quercetin), and two monoterpenes (1,8-cineole and α-pinene), which vary in chemical class, lipophilicity, and primary detoxification pathways (Tables 1 and 2). Although the specific pathways used by deer for these four PSMS are yet unknown, using this suite of PSMs allowed us to examine the effects of different PSMs in mixtures that might have additive or synergistic effects. All four PSMs can have antioxidant, anti-inflammatory, and anti-microbial functions (Cosme et al. 2020; Qi et al. 2022; Pries et al. 2023), which can benefit or harm the host or microbial taxa. All PSMs were also expected to compete to some degree for glucuronidation pathways that can be limited by substrate. Our criteria for selecting the chemicals were that (1) they are common in plants consumed by deer, (2) have evidence of deterring feeding by deer or other wild herbivores, (3) are available commercially.

**Table 2:** Diets fed to captive mule deer (*Odocoileus hemionus*) and white-tailed deer (*Odocoileus virginianus*) in controlled feeding trials including the plant secondary metabolites (PSM) added to a basal diet, their chemical class, ratio of chemicals mixed to equal 2.5% of the diet by mass, and trial start date

PSM mixture	Chemical class	PSM ratio	Trial start date
1,8-cineole/α-pinene	Monoterpene only	50:50	7/12/23
Salicin/α-pinene	Phenolic only	50:50	9/15/23
Quercetin/salicin	Phenolic/monoterpene	50:50	10/17/23
Quercetin/α-pinene	Phenolic/monoterpene	50:50	11/7/23
Quercetin/salicin/1,8-cineole/α-pinene	Phenolic/monoterpene	25:25:25:25	12/3/23

Within the general phenolic class, we selected salicin, which is a phenolic glycoside (Berger et al. 2012, 2015) and is found in willows (*Salix* spp.), poplars (*Populus* spp.), and other deciduous plant species that deer consume (Raskin 1992; Mahdi 2014; Hull et al. 2020). Like most phenolics, salicin has relatively low lipophilicity and depending on the host physiology, may require biotransformation before it can be absorbed by the animal (Pass and Foley 2000; Mahdi 2014) and may also be absorbed, but not detoxified by the host (McLean et al. 2001). The major biotransformation pathways for salicin include hydrolysis and conjugation with GA (Table 1, Fötsch et al. 1989, McLean et al. 2001). Transformation can include cleavage of glucose from the molecule, which could also be absorbed by the host or used as a substrate for microbes (Julkunen-Tiitto And Meier 1992; McLean et al. 2001; Mahdi 2014). Salicin can inhibit Phase I enzymes (Gomes et al. 2021), which can alter absorption and detoxification of co-ingested PSMs and can be activated by microbes to create intestinal homeostasis (Kuziel et al. 2025). Salicin is known to reduce intake in mammalian herbivores (Marsh et al. 2006b; DeGabriel et al. 2009b; Pass and Foley 2000; Tahvanainen et al. 1985).

Our second phenolic was quercetin, a flavonone found in willows and other deciduous plants consumed by deer (Riddick 2021; Bai et al. 2025), with a more complex structure and higher molecular weight than salicin. In clinical trials, quercetin can inhibit several CYP enzymes in host cells and microbes (Deodhar et al. 2020), reducing the rate of detoxification of other PSMs (Elmeliegy et al. 2020), but can also activate CYP enzymes (Patel 2004; Yu 2011). In domestic cows (*Bos* spp.) and rodents (Yeh et al. 2016) quercetin has been shown to primarily use the Phase II pathway requiring endogenous substrates including glucuronides and sulfates and methylation (Berger et al. 2012; Table 1). Rumen bacteria can break down quercetin into smaller, more absorbable phenolic acids (Sharma et al. 1981; Berger et al. 2012; Aldian et al. 2025). When ingested, quercetin can act as an antifeedant, reduce digestibility, and can be toxic in large concentrations (Berger et al. 2012; An et al. 2025).

Both monoterpenes we selected, 1,8 cineole (hereafter cineole) and α-pinene (hereafter pinene), are also found in woody plants that deer consume, especially in winter, such as sagebrush (*Artemisia* spp.), cedar (*Thuja* spp.), Douglas-fir (*Pseudotsuga menzeseii*), and western hemlock (*Tsuga heterophylla*, Wambolt 2001; Degenhardt et al. 2009; Ulappa et al. 2020). Both monoterpenes have a low molecular weight, relatively high lipophilicity, can pass across the blood brain barrier, and can inhibit CYP enzymes (Pass and McLean 2002; Green et al. 2004, 2006; Elmeliegy et al. 2020; Deodhar et al. 2020). Cineole has been shown to reduce intake in wild (Boyle et al. 2005; Marsh et al. 2006b; DeGabriel et al. 2009b; Shipley et al. 2012) and domestic (Dziba et al. 2006) mammalian herbivores. Pinene reduced intake in both deer species used in this study (*Odocoileus* spp., Staudenmeier et al. 2022) and other wild (Torregrossa et al. 2011; Nobler et al. 2019) and domestic mammalian herbivores (Estell et al. 2014).

Metabolic pathways of 1,8 cineole, a bicyclic monoterpene, are relatively well-known in some small hindgut fermenters (e.g., koalas [*Phascolarctos cinereus*], brushtail possums, wild and domestic rabbits, Miyazawa et al. 1989; Boyle et al. 1999, 2000; Shipley et al. 2012). Detoxification in these species proceeds primarily through serial oxidation, and cineole metabolites can be conjugated with GA (Table 1), with the extent of conjugation decreasing with prior metabolism from Phase I that increases polarity (i.e., oxidation, Boyle et al. 2000b; Shipley et al. 2012). Dietary specialists can oxidize 1,8 cineole to a greater extent than generalists (Pass et al. 2001), whereas dietary generalists use energetically expensive conjugation to a greater extent than dietary specialists (Boyle et al. 1999, 2000a; Shipley et al. 2012). Pinene is a bicyclic monounsaturated monoterpene, and its detoxification pathways have been explored in domestic and wild mammalian herbivores (Ishidax et al. 1981; Scramm et al. 2024; Southwell et al. 1980). Similar to 1,8 cineole, oxidation is the primary pathway for pinene and pinene metabolites can be conjugated with GA (Table 1). The dependence on specific pathways and composition of metabolites of pinene differs among animal species and degree of dietary specialization (Scramm et al. 2024).

Less is known about the specific metabolic pathways of monoterpenes in wild ruminants, but both cineole and pinene were degraded to some extent in rumen fluid from sheep and goats (Malecky et al. 2009a, b; Poulopoulou and Hadjigeorgiou 2021) and the microbial community composition differed on goats fed high pinene diets, with a reduced abundance of *Ruminococacceae* (Seidel 2020). However, based on previous work indicating that deer browse on a wide variety of woody plants that produce a diversity of phenolic and monoterpene PSMs (Wambolt 2001; Degenhardt et al. 2009; Berry et al. 2019; Ulappa et al. 2020), we would expect deer to reflect what has been found in other generalists hindgut browsers (Pass and Foley 2000; Marsh et al. 2006b; Kohl and Dearing 2016; Schramm et al. 2024) and rely on both oxidation and conjugation with GA when consuming both cineole and pinene.

### Measuring Responses to PSMs

To test predictions about the effects of PSM mixtures on behavior (DMI, PSMI), physiology (GA), and microbial communities (community richness and diversity) in deer, we conducted a series of feeding trials with six female deer of two congeneric species (three mule deer and three white-tailed deer). Deer species were grouped together for this study because we assumed they would respond similarly to the mixed diets because they are similar in size, physiology, and nutritional requirements and co-occur along a broad zone roughly following the Rocky Mountains in the western North America (Geist 1998; Berry et al. 2019; Staudenmaier et al. 2021). They consume similar diets where sympatric (Berry et al. 2019) and their intake declined similarly with increasing dietary pinene in previous experiments (Staudenmaier et al. 2022; Anderson et al. 2025). All deer were raised and housed under identical environmental conditions at the Wild Ungulate Facility (WUF) located at Washington State University in Pullman, WA, to ensure similar exposure to microbes, diets, and learning experiences. One fawn was born at WUF in 2009 of parents that had been captured from the wild as neonates, and the remaining five were wild-born and acquired from wildlife rehabilitators in 2014 and 2015 when they were less than 3 days old. Fawns were bottle-fed with deer milk replacer (Fox Valley DayOne^®^ 30/40, Animal Nutrition Inc., Lake Zurich, IL, USA) following established feeding schedules that mimic natural feeding and growth patterns of dam-raised fawns (Parker and Wong 1987). Fawns were also offered a balanced grain-alfalfa pelleted ration, pasture grass, alfalfa, and soil within the first weeks of life to help stimulate growth and development of the rumen and GI tract. As adults, all deer were housed together and exposed to a variety of natural forages with PSMs both as supplementary browse at WUF and in field experiments during which they spent weeks to months foraging at field sites over multiple years for other research projects (Wagoner et al. 2013; Hull 2018; Berry et al. 2019). All deer ranged from 10 to 16 years of age and 50 to 88 kg in body mass during the experiments.

During the PSM mixture trials, deer were housed individually in 1.9 × 1.9 m covered outdoor digestion crates, constructed with chain link fence, rubberized flooring, and deer-safe materials. Deer had been trained to use the digestion crates during previous studies (e.g., Staudenmaier et al. 2022) and had been exposed to each PSM individually mixed with the basal diet in preliminary trials that lasted several days. All procedures were approved by Washington State University Institutional Animal Care and Use Committee (IACUC; protocol # 6727). We conducted trials with all animals at the same time with diets in the same sequence. Each PSM mixture trial began with a two-day acclimation on the PSM-free basal diet. The basal diet was a custom-made and completely balanced grain-alfalfa deer pellet consisting of 29.9% neutral detergent fiber, 4.0% acid detergent lignin, resulting in 17.7 kJ/g of gross energy and 18.1% crude protein (Staudenmaier et al. 2022). Following the PSM-free acclimation, we fed all deer one of five PSM mixture diets for five days. These diets included two same-class PSM diets (phenolics – salicin/quercetin and monoterpenes—cineole/pinene), two different-class PSM diets (salicin/pinene and quercetin/pinene), and one mixture of all four PSMs (4-way mixture, Table 2). PSMs were mixed in ratios so that each diet had a total of 2.5% PSMs. The 2.5% concentration was based on the typical concentration of single PSMs in natural forage consumed by wild herbivores (Palo 1984; Stark et al. 2008; Nobler et al. 2019) to provide doses that were biologically meaningful and could be tolerated. PSM diets were created by hand mixing the PSM mixture with the PSM-free basal diet. Salicin and quercetin (BenchChem, Pasadena, CA United States) are distributed as a powder, requiring us to add a small amount of water to coat the pellets. We purchased cineole and pinene in its essential oil form (Sigma-Aldrich Canada, Oakville, Ontario, Canada) so it was readily absorbed into the pellets.

Each day that we fed the PSM-free (acclimation) and PSM-mixture diets, we weighed the amount of food offered to the deer *ad libitum* and collected and weighed uneaten food (orts) the following morning. Feces were collected on a mesh screen under the floor and weighed daily and urine was funneled into a closed container under the crate and volume measured. Food offered, orts, and feces were corrected for % dry matter by weighing a sample fresh and then again after drying it at 100 °C for 24 h. On days 3 (PSM-free diet), 5, and 7 (PSM-mixture diet) of the experiment, we collected and immediately froze (−20 °C) fecal samples for microbial analysis and urine samples for GA analysis, representing values for the previous two days’ diet. Animals were removed from a trial if they ate < 500 g of food for two consecutive days, and if an animal left the trial before day 7, we collected fecal and urine samples on the last full day of the trial. One white-tailed deer was consistently removed on day 5 from the cineole/pinene trial, the salicin/pinene, salicin/quercetin trial and another white-tailed deer was removed on day 5 of the 4-way mixture trial. We wore nitrile gloves for collecting samples, changing them between each sample and each individual. Fecal samples were placed into re-closable plastic bags and urine collected in sterile glass vials. We calculated daily dry matter intake (DMI, g/day) as dry food offered less orts, and PSM intake (PSMI, g PSM/day) as the product of DMI and PSM concentration of the diet (0 or 2.5%). For analysis, we averaged DMI and PSMI across the 2 days animals were fed the PSM-free and the 5 days they were fed the PSM diet. Trials were conducted at least 2 weeks apart, allowing deer the chance to recover from each exposure of the previous PSM mixture (Table 2).

### Microbial Analysis

We analyzed fecal samples from each deer for each PSM-free and each 2.5% PSM mixture diet for microbial composition and diversity using a 16 s rRNA approach (Combrink et al. 2023). Using fecal samples as a proxy for GI microbial sampling is a common, noninvasive method for herbivores including wild ruminants (Combrink et al. 2023; Anderson and Shipley 2025; Buchanan et al. 2024). Samples were extracted using the Qiagen RNEasy PowerMicrobiome Kit with manufacturer recommendations. The amount of DNA in each sample was measured using a Qubit (Invitrogen, Inc., Carlsbad, CA), and 5 µl of each DNA suspension was amplified by polymerase chain reaction (PCR) using a MasterCycler thermocycler (Eppendorf, Westbury, NY). The 16 S rRNA gene was amplified with the 806 F and 926R primers that target the V4-5 region. Negative controls went through extraction and library prep were included in the sequencing but did not produce any sequence data. After extraction, samples were sequenced on an Illumina MiSeq Platform, using 2 × 150 bp paired-end sequencing. Samples were sequenced using a 600-cycle MiSeq Reagent Kit v3 (Illumina^®^) in the presence of 25% PhiX DNA. Following extraction, samples were processed using the software *Quantitative Insights Into Microbial Ecology* (QIIME2; Boylen et al. 2019) pipeline, beginning with demultiplexing and removing samples that did not amplify, removing sequences that had less than 300 bp. We then denoised and dereplicated samples using the *Divisive Amplicon Denoising Algorithm* (DADA2) module within QIIME2 and removed sequences that had less than 300 bp. Unique amplicon sequence variants (ASVs) were generated using DADA2 and assigned a taxonomy using the SILVA 138 ribosomal RNA database. ASVs that could not be assigned to phylum were removed. Downstream analysis was done in R v 4.0.735. We imported QIIME2 readable files (*.qza) into R using the package ‘qiime2R’ v0.99 (Bisanz 2024). We then used the ‘*phyloseq*’ package (McMurdie and Holmes 2013) to remove non-bacterial, mitochondrial, and chloroplast ASVs, and then remove the negative controls, once it was determined there was no contamination.

### Glucuronic Acid Assays

 Urine samples collected during the feeding trials were analyzed for GA following the methods outlined in Mangione et al. (2001) using a colorimetric assay. Samples were first thawed, mixed with a solution of borax (sodium tetraborate) and sulfuric acid, and heated to 100 °C for 10 min to create a colorimetric reaction. A standard curve was used to estimate GA quantity from absorbance values read using a spectrophotometer at 520 nm (Sigma-Aldrich Canada, Oakville, Ontario, Canada, 50–370 μm range). We determined the total amount of GA produced per day by each animal by multiplying the GA concentration (mM/ml) by urine volume on the sampling day (ml). To calculate the amount of GA excreted relative to PSMs ingested (mM GA/g PSM/day), we divided the GA produced per day (mM GA/day) by the amount of PSM consumed (g/day) by the deer.

### Statistical Analyses

We calculated alpha diversity of the fecal microbial community, which quantifies diversity within individual samples and can be compared across sample groups, using two metrics: overall species richness (hereafter referred to as richness) and the Shannon-Weiner Diversity Index (hereafter referred to as the Shannon Index). We characterized richness from the number of microbial sequences per sample. We calculated the Shannon Index from the number of unique reads and their proportional abundance in the sequence, using the ‘phyloseq’ package (McMurdie and Holmes 2013). In our preliminary analysis of these data, we found that the microbiome of one animal on the 4-way mixture trial was dominated by *Listeriaceae*, which includes pathogenic species that are associated with listeriosis, a bacterial infection that can cause diarrhea and other GI symptoms (Alam et al. 2021) in ungulates. Because of the potential for this disease to influence the results of our study, we removed the animal from the 4-way mixture trial before analysis but also provided the results of that analysis in the Appendix (Figure 7).

To determine the effects of PSM mixtures on the DMI, PSMI, GA, and microbial community diversity, we calculated the net change between the 2.5% PSM and PSM-free diet that preceded it by subtraction for each response variable and animal. Using this metric allowed us to control for any possible carryover effect from a previous trial. To address our first hypothesis (P1) that consuming a diet of 2.5% PSM mixture would influence behavior, physiology, and microbial communities of deer, we compared the net change in each response variable (DMI, PSMI, GA, microbial richness and Shannon Index) with the null hypothesis of no change (0) using a one-sample t-test. Next, we compared the net change in each response variable among the five PSM mixtures (P2, P3) using Linear Mixed Effect Models (LMER) with individual animal as the grouping variable using the ‘lme4’ and ‘lmerTest’ packages in R (Kuznetsova et al. 2013; Bates et al. 2017). Using individual animal as a grouping variable allowed us to control for characteristics of individuals (e.g., behavior, body mass) and deer species. We reran the LMER with different PSM diets as the reference variable so we could compare diets.

Finally, we calculated beta diversity of microbial communities to compare their dissimilarity between diet trials, creating a distance matrix between all pairs of samples. To quantify the beta diversity of the microbiome, we first filtered and clustered sequences into amplicon sequence variants (ASVs). ASVs were used to reconstruct phylogenies, allowing us to calculate microbial beta diversity and relative abundances (Combrink et al. 2023). We then transformed the read counts of our non-rarefied data to relative abundance by dividing the number of reads for each taxon within a sample by the total number of reads for that sample. We performed a Permutational Multivariate Analysis of Variance (PERMANOVA) to compare Bray-Curtis distances among diets, using the ‘*adonis2*’ function in the vegan package v2.6-4, ordinations were then replotted using just the centroids with the arrows indicating the direction of change considering mixtures. (Oksanen et al. 2024). We calculated the difference in relative abundances for the top 20 families identified in each chemical by subtracting microbial abundance on the PSM-free diet from the abundance on the 2.5% PSM diet. We then compared the change in the relative abundance of selected microbial families to 0 and between chemicals using parametric ANOVAs, and the means were separated using the Tukey test.

## Results

**Fig. 2:**
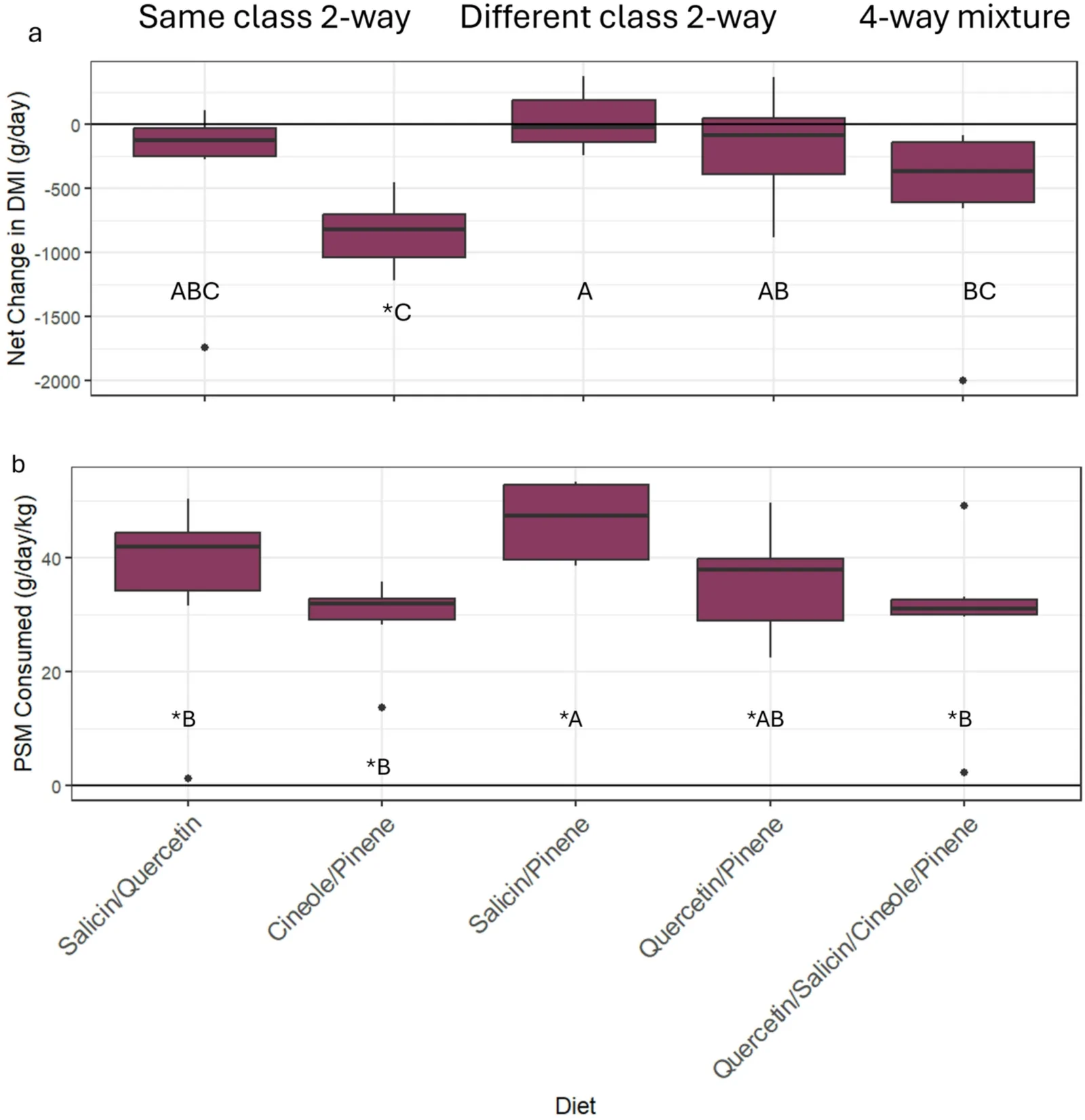
Differences in the mean voluntary dry matter (DM) intake (**a**) and plant secondary metabolite (PSM) intake (**b**) per day by mule deer (*Odocoileus hemionus*) and white-tailed deer (*Odocoileus virginianus*) when fed a basal diet containing 2.5% of a mixture of purified PSMs compared to a PSM-free basal diet fed just prior. Higher means indicate a greater positive or less negative effect of 2.5% PSM. Cineole and pinene refer to 1,8-cineole and α-pinene respectively. Asterisks denote significant differences between 2.5% and 0% concentration for each PSM, and different capital letters denote significant differences among PSM mixtures (α = 0.05)

Overall, behavioral responses in deer consuming diets with 2.5% PSM mixtures were less consistent than physiological responses predicted (P1, Fig. 1). Behaviorally, only the same-class monoterpene diet significantly reduced voluntary DMI compared to the PSM free diet (mean net change < 0) (overall *F* = 2.26, *P* = 0.09, cineole/pinene *F* = −2.59, *P* = 0.02, Fig. 2a). However, because all animals ate at least some amount of each PSM diet, PSMI was greater than 0 for all PSM diets mixtures (*F* = 2.97, *P* = 0.04). The effect of consuming PSM mixtures of the same and different chemical classes only partially supported our predictions (P3, Fig. 2) based on the detoxification limitation hypothesis (Fig. 1). As predicted, deer consumed more when offered both 2-way different-class mixtures than the same-class mixture of two monoterpenes. However, the same-class mixture of two phenolics did not differ from any other diet mixture. Contrary to our expectation, they also ate more of one of the 2-way different-class mixtures (salicin-pinene) than the 4-way different class mixture (Fig. 2a). Similarly, PSMI was higher for one 2-way different-class mixture (salicin-pinene) than both same-class mixtures and the 4-way different class mixture.

**Fig. 3:**
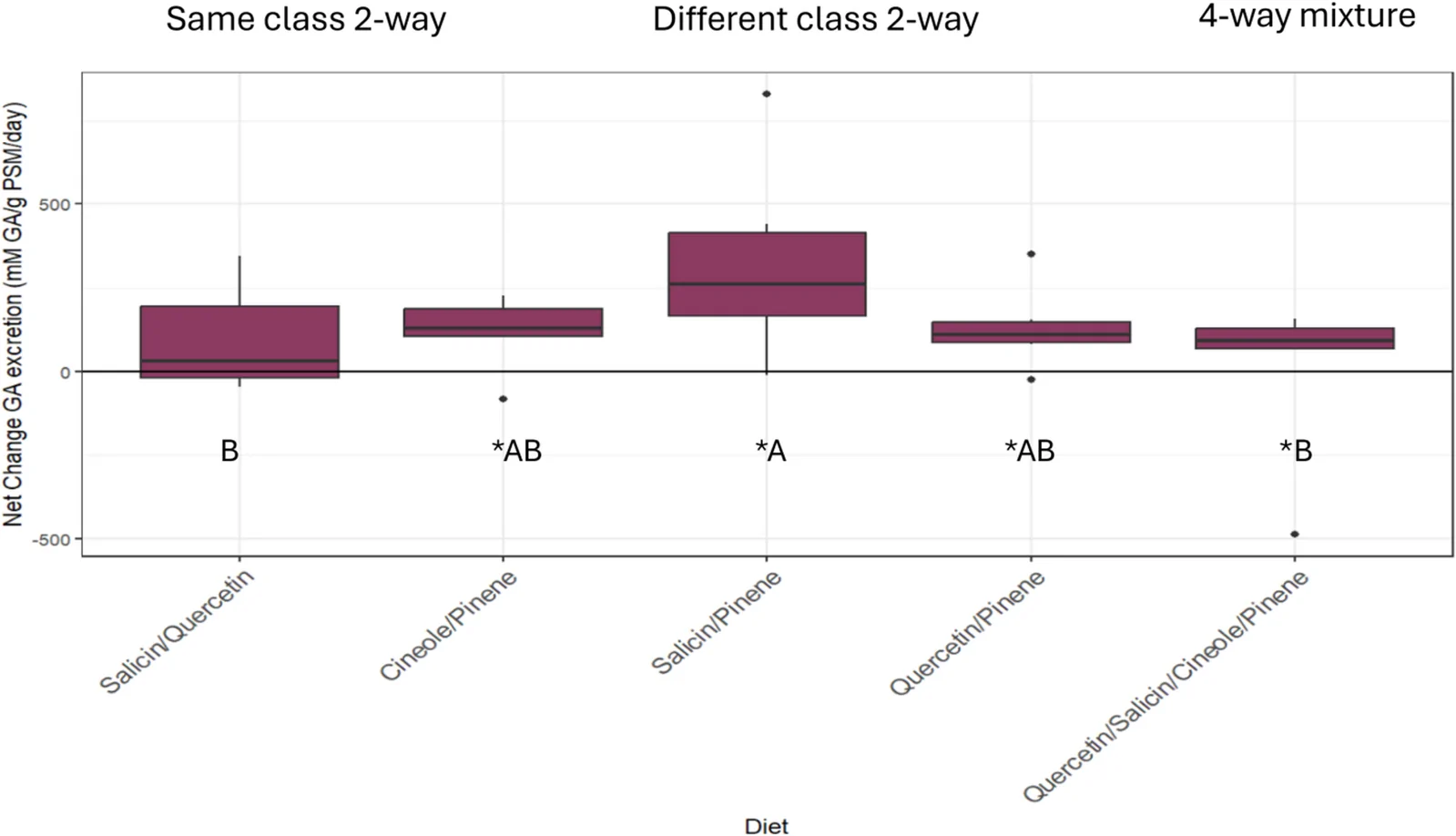
Differences in the mean glucuronic acid (GA) excretion (mM GA/g PSM/day) by mule deer (*Odocoileus hemionus*) and white-tailed deer (*Odocoileus virginianus*) when fed a basal diet containing 2.5% of a mixture of purified PSMs compared to a PSM-free basal diet fed just prior. Higher means indicate a greater positive or less negative effect of 2.5% PSM. Cineole and pinene refer to 1,8-cineole and α-pinene respectively. Asterisks denote significant differences between 2.5% and 0% concentration for each PSM, and different capital letters denote significant differences among PSM mixtures (α = 0.05)

Despite the limited behavioral response, physiological responses through GA excretion increased significantly on all 2.5% PSM mixtures except the same-class phenolic diet (*F* = 2.70, *P* = 0.05, Fig. 3). Although our physiological prediction was that net GA production per unit of PSM intake would be highest on diets with higher lipophilic PSMs (P2), GA excretion on the same-class monoterpene mixture did not differ from other mixtures (*P* = 0.32). However, the different-class salicin-pinene mixture had significantly higher GA than the phenolic same-class mixture (*P* = 0.046) and the 4-way different-class mixture (*P* = 0.012, Fig. 3).

**Fig. 4:**
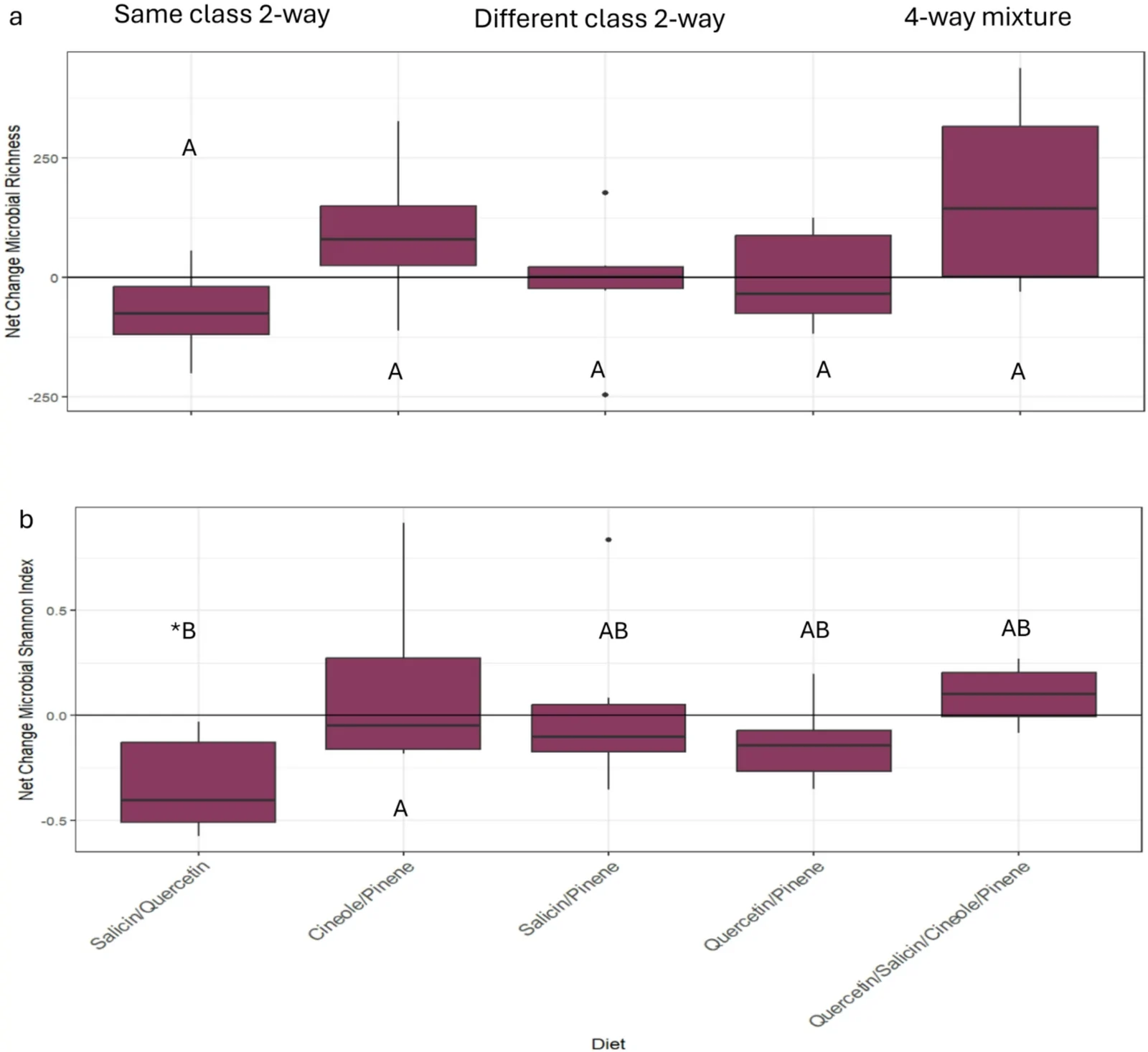
Differences in the mean microbial richness (number of taxa, **a**) and microbial Shannon-Weiner Index (**b**) in feces produced by mule deer (*Odocoileus hemionus*) and white-tailed deer (*Odocoileus virginianus*) when fed a basal diet containing 2.5% of a mixture of purified PSMs compared to a PSM-free basal diet fed just prior. Higher means indicate a greater positive or less negative effect of 2.5% PSM. Cineole and pinene refer to 1,8-cineole and α-pinene respectively. Asterisks denote significant differences between 2.5% and 0% concentration for each PSM, and different capital letters denote significant differences among PSM mixtures (α = 0.05)

**Fig. 5:**
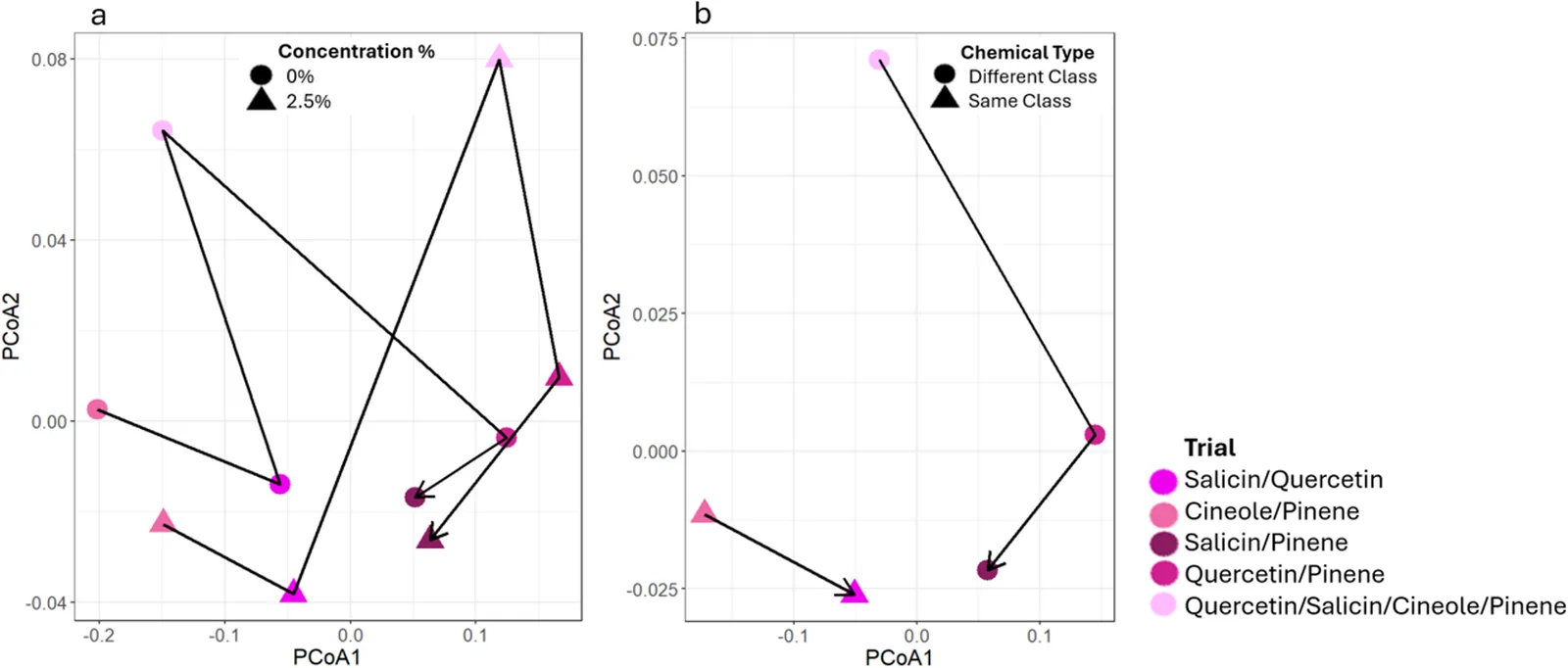
Ordinations of microbial beta diversity of feces produced by mule deer (*Odocoileus hemionus*) and white-tailed deer (*Odocoileus virginianus*) (**a**) when fed diets containing mixtures with a total concentration of 2.5% concentration purified plant secondary metabolites (PSMs, phenolics: salicin and quercetin, monoterpenes: 1,8 cineole and α-pinene) compared to PSM-free diets (0% PSM), and (**b**) when consuming mixtures of the same chemical class (both phenolics, both monoterpenes) compare to mixtures of different chemical classes (one phenolic and one monoterpene or two of each). Beta diversity ordinations were based on a PCoA using Bray-Curits distance metrics, ordination were then replotted using just the centroids with the arrows indicating the direction of change considering mixtures

Finally, PSMs had nuanced effects on overall gut microbial diversity and composition. PSMs did not significantly alter gut microbial richness (*P* = 0.063) or diversity (*P* = 0.12) on any mixture at 2.5%, except that microbial richness decreased in the same-class phenolic trial (*F* = 2.49, *P =* 0.02, Fig. 4a, b). Fecal microbial richness did not differ significantly among PSM mixtures (*P* = 0.046, Fig. 4a). However, consistent with the prediction that low lipophilic phenolics would interact more with microbes with specific biotransformation capacity (P2), the same-class phenolic diet had lower microbial Shannon Index than did the same-class monoterpene diet (Fig. 4b). Across PSM mixtures, a 2.5% concentration of PSM significantly influenced the beta diversity of the GI microbial community (PERMANOVA R^2^ = 0.20, F = 2.37, *P* = 0.04, Fig. 5a). Dispersion of beta diversity differed between the 2-way phenolic mixture and both 2-way different class mixtures (salicin/pinene, *P* = 0.008, quercetin/pinene, *P* = 0.03). The 4-way different-class mixture had a different dispersion than one of the 2-way different-class mixtures (salicin/pinene, *P* = 0.05, Fig. 5b).

**Fig. 6:**
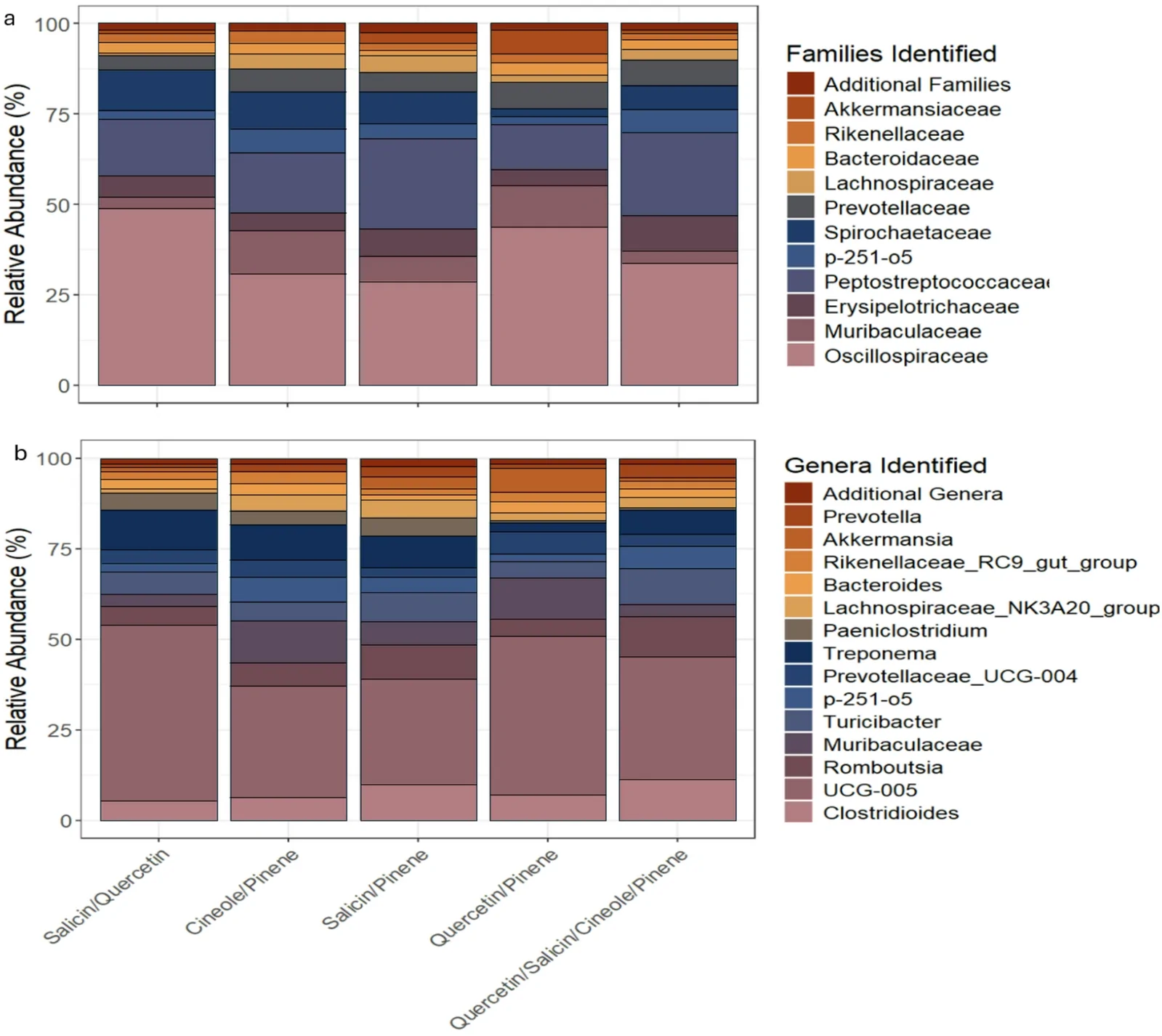
Top 20 bacterial families (**a**) and genera (**b**) identified from feces produced by mule deer (*Odocoileus hemionus*) and white-tailed deer (*Odocoileus virginianus*) when fed a basal diet containing 2.5% concentrations of mixture of purified PSMs (salicin, quercetin, 1,8 cineole, α-pinene) compared to a PSM-free basal diet. Families and genera are ordered from most to least abundant from bottom to top. One animal was removed from the 4-way diet because it had extremely high abundance of the bacteria *Listeria*, a known pathogen (Appendix A)

**Fig. 7:**
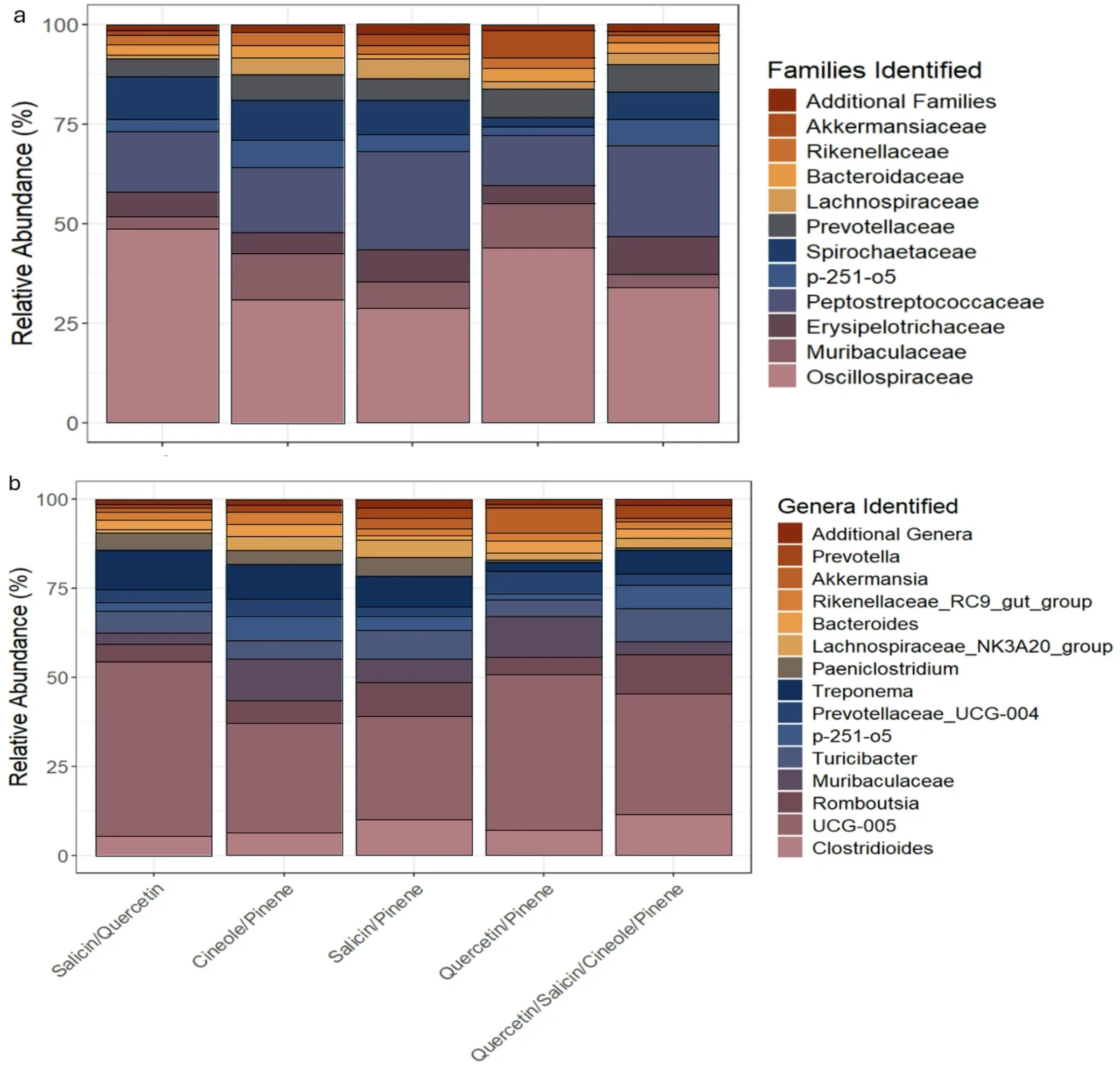
Top 20 bacterial families (**a**) and genera (**b**) identified from feces produced by mule deer (*Odocoileus hemionus*) and white-tailed deer (*Odocoileus virginianus*) when fed a basal diet containing 2.5% concentrations of mixture of purified PSMs PSMs (salicin, quercetin, 1,8 cineole, α-pinene) compared to a PSM-free basal diet. Families and genera are ordered from most to least abundant from bottom to top. An individual animal that had a high abundance of *Listeriaceae* in the quercetin/salicin/cineole/pinene mixture trial is included

PSM mixtures also influenced aspects of the composition of microbial communities. Three members of the top 20 families identified were only present when deer consumed the PSM diets (Fig. 6.), including *Akkermansiaceae* and some genera in the *Prevotellaceae* family (Fig. 6), that were found in all of the PSM mixture diets. Several genera of *Prevotellaceace*, and *Oscillospiraceae*, were only detected when deer consumed the PSM-free diet (Fig. 6). The microbial family *Oscillospiraceae* (*P* = 0.01) was less abundant between when consuming 2.5% than 0%, and *Peptostreptococcaceae* (*P* = 0.01) was more abundant when consuming the same-class phenolic diet than the PSM-free diet (Fig. 6). The net change in abundance between the PSM and PSM-free diets for *Oscillospiraceae* was lower (net decrease) in the same class monoterpene diet (µ = −4.62, SE = 1.85) than one of the 2-way different-class diets (salicin/pinene, net increase, µ = 1.21, SE = 0.97, *P* = 0.03, Fig. 6). The effect of PSM mixtures on *Peptostreptococcaceae* abundance was lower (net reduction) on the 4-way different class diet than both same-class diets (net increase, monoterpene: µ = 3.42, SE = 0.47, *P* = 0.05; phenolic: µ = 6.04, SE = 0.62, *P* = 0.003, Fig. 6).

We did not detect any of the microbial genera in deer fecal samples that have been previously associated with biotransformation of terpenoids in mammals (Table 3). However, we did detect three families with one or more genera that have been found to detoxify phenolics. *Lactobacillaceae* was one of the most abundant families identified in the 4-way different-class diet at 28%. *Lachnospiraceae* had a 5% abundance in the quercetin/salicin and 4% abundance in the quercetin/pinene trial. *Prevotellaceae* and *Peptostreptococcaceae* was found in all trials at 0.8 to 4% abundance and 0.4 to 11%, respectively.

**Table 3:** Microbial families and genera in the gastrointestinal tract that have been implicated in detoxifying phenolics and monoterpenes in mammalian herbivores. Chemicals used in the reported experiments are in bold

Chemical Class	Chemical	Family	Genus	Citation
Phenolics				
	Tannins, Phenolic acids	*Bacillaceae*		(Rogowska-van Der Molen et al. 2023; Kohl et al. 2016)
			*Bacillus*	
		*Bacteriodaceae*		(Lauterwein et al. 1995; Sundset et al. 2007)
			*Bacteroides*	
	Anthocyanins	*Bifidobacteriaceae*		(Faria et al. 2014; Rogowska-van Der Molen et al. 2023﻿)
			*Bifidobacterium*	
	Polyphenols	*Eggerthellaceae*		(Koppel et al. 2018)
			*Eggerthella*	
	Tannins	*Enterococcaceae*		(Kohl et al. 2018)
			*Enterococcus*	
	Tannins, Anthocyanins	*Lactobacillaceae*		(Shimada et al. 2006; Faria et al. 2014; Kohl and Dearing 2016)
			*Lactobacillus*	
	Phloroglucinol	*Lachnospiraceae*		(Patel et al. 1981)
			*Coprococcus*	
	Tannins	*Pectobacteriaceae*		(Kohl et al. 2016)
			*Escherichia*	
	Tannins	*Prevotellaceae*		(Li et al. 2015)
			*Prevotella*	
	**Salicin**, Camphor	*Pseudomonadaceae*		(Marmulla and Harder 2014; Dahal et al. 2023; Rogowska-van Der Molen et al. 2023)
			*Pseudomonas*	
		*Rumioncocacceae*		(Varel and Jung 1986)
	**Salicin**	*Yersiniaceae*		(Dahal et al. 2023; Rogowska-van Der Molen et al. 2023)
			*Rahnella*	
Terpenes				
	**Cineole**	*Enterobacteriaceae*		(Marmulla and Harder 2014)
			*Citrobacter*	
	Limonene	*Pectobacteriaceae*		(Marmulla and Harder 2014)
			*Enterobacter*	
	**Pinene**	*Pseudomonadaceae*		(Marmulla and Harder 2014)
			*Pseudomonas*	
	**Pinene**	*Yersiniaceae*		(Dahal et al. 2023; Rogowska-van Der Molen et al. 2023)
			*Rahnella*	
	Menthol, Eucalyptol	*Zoogloeaceae*		(Hylemon and Harder 1998; Marmulla and Harder 2014)
			*Thauera*	

## Discussion

Mule and white-tailed deer responded to mixtures of PSMs with more consistent physiological responses than through behavioral and microbial responses (P1). Although PSM mixtures had some influence on DMI and microbial communities, we found that an increased excretion of GA was the most consistent effect of dietary PSM mixtures. This result suggests that at the concentration we fed, the animal’s detoxification physiology generally was able to eliminate toxic PSMs fast enough to allow the deer to maintain their food intake and for the microbial community to remain functionally stable. Overall, physiological and microbial responses supported predictions of lower host absorption and higher microbial interactions based on lipophilicity of PSMs consumed (P2). We found a greater influence of monoterpenes on intake and GA excretion, but a lesser effect on microbial diversity, than phenolics. In addition, the same-class phenolic diet was the only diet that did not result in increased GA suggesting lower absorption of the phenolic mixture. Importantly, individual microbial taxa known to detoxify phenolics were more abundant when animals ate mixtures containing phenolics suggesting more microbial interactions with phenolics than monoterpenes. Finally, results only partially supported predictions of the detoxification limitation hypothesis that intake of any one PSMs is limited by the capacity of detoxification pathways. and this limitation could be mediated by changes in intake, depletion of detoxification cofactors, and biotransformation of PSMs by microbial communities (P3). Based on this hypothesis, we expected that consuming mixtures of PSMs that primarily use different detoxification pathways (e.g., a phenolic and a monoterpene) and presumably different microbes would allow for more efficient detoxification (lower GA) and elimination than same-class mixtures, thus would have greater voluntary intake, reduced GA excretion, and more diverse microbes. In support, deer consumed more when offered different-class PSM mixtures than the same-class monoterpene mixture. Contrary to the hypothesis, deer did not eat more when offered the most diverse diet of four different PSMs. Furthermore, glucuronidation and microbial communities were more strongly influenced by phenolics rather than the mixture of PSM classes. Our findings highlight the complexity of interactions between behavior, physiology, and microbial communities when foregut fermenting herbivores consumed PSM mixtures.

Although GA excretion increased for almost all the PSM mixtures we offered, the behavioral response of deer was modest. The minimal response in DMI to PSMs is not altogether surprising for several reasons. First, of the possible strategies that an animal can use to minimize the deleterious effects of PSMs, the behavioral pathway may be the most costly and last defense employed. Although animals experience energetic and other nutritional costs when detoxifying and eliminating PSMs, these costs often may not exceed the gain in energy and nutrients animals would acquire from consuming more food. Sorensen et al. (2005a) found that when given increasing amounts of a high PSM juniper (*Juniperus* spp.) diet to woodrats, the animals reduced activity before they reduced food intake, ensuring that they could continue to acquire energy needed to maintain body condition and to detoxify the PSMs. Furthermore, this change in activity occurred gradually over a three-week period, suggesting that animals might experience the physiological costs associated with detoxification at a relatively slow rate (Sorensen et al. 2005a). The gradual energetic adjustment to PSM diets was also reported by Malecky et al. (2009a, b) when Saanen goats (*Capra aegagrus hircus*) were offered increasing concentrations of a monoterpene mixture, they found no effect on DMI over their six-week trial. The trade-off between reducing food consumption and bearing the costs of detoxification likely depends, in part, on the nutritional quality of the food consumed (Malecky et al. 2009a, b; Nersesian et al. 2012; Staudenmaier et al. 2022; Nemli et al. 2024). In our experiments, PSMs were added to a basal diet high in protein and digestible energy, which prevented depletion of substrates and co-factors that could limit detoxification and might have reduced the fitness value of minimizing PSM intake to avoid detoxification costs. Mammals often ingest more PSMs if supplemented with protein, potentially because protein is required to synthesize enzymes used in detoxification and to repair tissues damaged by toxins (Campbell et al. 2007; Pfister et al. 2012; Au et al. 2013; Moore et al. 2015).

A second potential reason for the modest behavioral response to dietary PSM mixtures is that we chose to feed deer a total concentration of 2.5% to reflect concentrations of those PSMs normally found in natural forage consumed by wild deer (Palo 1984; El-Shazly et al. 2012; Mahdi 2014; Lasinskas et al. 2024). Although some studies used the same or lower concentrations of purified PSMs mixed with basal diet (Boyle et al. 2000a, b; McLean et al. 2008), others used artificially high concentrations that caused substantial reductions in voluntary intake (e.g., 5–15%, DeGabriel et al. 2008; Nersesian et al. 2012; Shipley et al. 2012; Kohl et al. 2016; Staudenmaier et al. 2022). Voluntary intake is expected to decline as the concentration of circulating toxins exceeds an animal’s physiological threshold for detoxification (McLean et al. 2008; Forbey et al. 2018). Thus, our findings suggest that for most of the PSM mixtures we offered, deer were likely able to detoxify and eliminate them at the 2.5% concentration without reaching their physiological threshold. The deer also might have avoided exceeding thresholds in PSM plasma concentration and thus detoxification capabilities by consuming the same total amount but spreading their feeding bouts in small meals throughout the day (Sorensen et al. 2005b; Marsh et al. 2006b; McLean and Duncan 2006; Torregrossa and Dearing 2009). Although our limited sample size did not allow us to statistically compare intake, GA excretion, and microbial responses between deer species, some evidence suggests that mule deer might be slightly more efficient in detoxifying monoterpenes and phenolics than white-tailed deer. When foraging together in the same plots, mule deer consumed more deciduous and evergreen shrubs than did white-tailed deer, and when fed increasing amounts of pinene, mule deer excreted less GA than white-tailed deer. Improving our understanding of responses of wild ruminants and differences between species would not only benefit from larger sample size, but also from a wider range of PSM concentrations across longer time periods (e.g., 2 weeks), that would elucidate effects of longer term induction of CYPs with PSM exposure (Sorensen et al. 2005; Marsh et al. 2006b) and adjustments in the microbial community (Kohl et al. 2014, 2016; Miller et al. 2014) coupled with data on adjustments to meal size in relation to circulating PSM exposure (Boyle et al. 2005).

Overall, the most notable differences among PSM mixtures across behavioral and physiological responses occurred between the 2-way phenolic and 2-way monoterpene diets, which generally conformed to our predictions (P2). We saw no reduction in DMI or increase in GA when deer consumed only phenolics, but the greatest reduction in DMI occurred when they consumed the 2-way monoterpene mixture. Because phenolics are hydrophilic, they may not be absorbed efficiently in the small intestine, which would reduce the need for extensive conjugation with GA by the host, thus lowering overall GA excretion (Bento-Silva et al. 2020). In contrast, lipophilic monoterpenes are quickly absorbed and transferred to the detoxification system in the liver, resulting in a higher endogenous energy cost as evidenced by higher GA excretion. These differences between behavioral and physiological responses to PSMs are consistent with other studies in which animals were fed monoterpene diets. For example, mule and white-tailed deer produced more GA per unit PSM consumed when consuming the monoterpene α-pinene than condensed tannins subclass of phenolics with a molecular weight at least three times higher than salicin and quercetin used in our study (Staudenmaier et al. 2022). White-tailed deer also had the highest GA: Creatine production when consuming balsam fir (*Abies balsamea*) that contained high levels of monoterpenes than a diet with mixed deciduous tree leaves (Servello and Schneider 2000), which typically contain more phenolics (Weunsch et al. 2025).

Responses of the deer’s GI microbial community to PSM mixtures might also play a complex role in their DMI and GA excretion. Although we expected a net reduction in microbial diversity when deer consumed any PSM mixture, the overall effect of PSMs on microbes was neutral except for when deer consumed the same-class phenolic mixture. This result suggests that the GI microbial community in concert with behavioral and physiological responses might play a larger role in degrading phenolics with presumed longer retention time before absorption than more lipophilic monoterpenes (Scogings et al. 2021), thus potentially explaining the lesser effects of phenolics than monoterpenes on DMI and GA in deer. The lower diversity on the same-class phenolic mixture compared to the same-class monoterpene mixture indicates phenolics might select against microbes that could offer resilience to the effects of PSMs and selection for microbes that use a PSM as their energy source or are able to degrade the PSM and avoid toxicity. A more diverse microbial community that fills more ecological niches in the host’s GI tract could reduce the costs of detoxification for the host through their own fermentation and metabolism (Beck and Gregorini 2020; Madi et al. 2020). In deer, we detected the presence of four microbial families that have been previously associated with degradation of phenolics in mammals (i.e., *Lactobacillaceae*, *Lachnospiraceae*, *Bacteriodaceae*,* Prevotellaceae*, Rodriguez-Daza et al. 2021; Gasaly and Gotteland 2022; Wang et al. 2022). These microbial families were only present when the deer consumed the same-class phenolic mixtures or certain mixtures comprised of phenolics. In contrast, we did not detect families known to degrade monoterpenes such as *Rahnellas pp*. (e.g. Pinene, Dahal et al. 2023; Rogowska-van Der Molen et al. 2023). In addition, of the most abundant families found in both PSM and PSM-free diets, *Oscillospiraceae* showed the greatest decrease on the monoterpene mixture and *Peptostreptococcaceae* showed the greatest increase on the phenolic mixture. Both are known to be involved in protein degradation and nitrogen recycling, but *Peptostreptococcaceae* can also degrade alkaloid PSMs (Rogowska-van Der Molen et al. 2023). Although alkaloids were not added to the diets fed in our experiments, *Peptostreptococcaceae* might include some genera that assist in detoxifying the PSMs that we used.

In addition to playing a role in detoxification, microbes can mediate the nutrition and health of the host. For example, byproducts of bacterial fermentation of PSMs can be used as nutrients by other microbes and the host (Duda-Chodak et al. 2015). For example, phenolic glycosides such as salicin are bound to glucose, which is removed through enzymatic hydrolysis by both the animal and microbes. The cleaved glucose can then be used by the host for their energetic needs including glucuronidation of PSMs instead of using endogenous glucose (Caldwell 1986; McArthur et al. 1991; Julkunen-Tiitto and Meier 1992; Mahdi 2014). Microbes can also be important bioindicators of overall health (Combrink et al. 2023; Ribas et al. 2023) which may further explain variable responses to PSMs. Although only speculative, the incidental finding that *Listeriaceae* and *Akkermansiaceae* were only identified on PSM diet offers some insights into the health of our deer. *Listeriaceae* is often associated with listeriosis, a bacterial infection that can cause diarrhea and other GI symptoms (Alam et al. 2021) and *Akkermansiaceae* has been linked to intestinal barrier integrity, metabolism, and gastrointestinal disease in laboratory animals and humans (Karcher et al. 2021; Pellegrino et al. 2023). In addition, the *Peptostreptococcaceae* that was more abundant when deer consumed phenolics contains the well-studied *Clostridioides difficile*, known for causing severe gastrointestinal infections and colon cancer in humans (Lessa et al. 2012). Although none of the individual deer showed any symptoms of illness or dysbiosis, these microbial taxa offer potential targets to predict host health (Bahrndorff et al. 2016; Mizrahi et al. 2021; Combrink et al. 2023), by taking advantage of other methods such as metabolomics to quantify changes in metabolites (Jeckel et al. 2022; Tan et al. 2024) and metagenomics to further quantify changes in microbial taxa (Caporaso et al. 2012; Callister et al. 2018; Chen et al. 2024).

Contrary to our predictions, our results provide only partial support for the detoxification limitation hypothesis (Freeland and Janzen 1974) that has been generally supported by other studies (Dearing et al. 2000; Marsh et al. 2006a; Sotka and Gantz 2013). We predicted that when deer were fed different-class mixtures, they would consume more, excrete less GA, and have higher microbial diversity by distributing the detoxification burden over more pathways in the host and microbes (P3, Marsh et al. 2006a). Deer voluntarily consumed more dry matter and PSM when consuming one of the 2-way different-class mixtures (i.e., salicin/pinene) than the same-class monoterpene mixture but also produced more GA on that mixture than on the phenolic mixture. Our finding that deer consumed the most dry matter and PSM on a 2-way different class diet (i.e., salicin/pinene) indicates that salicin may increase the deer’s tolerance to pinene. Salicylate, a precursor of salicin, has been shown to increase expression of other cytochrome P450 genes (Li et al. 2002) and enzyme activity (Ohtsuki et al. 2019). Both salicin (Marsh et al. 2006b) and pinene (Casabon and Pothier 2007; Bonnin et al. 2016) are known be detoxified using the GA conjugation pathway and animals gain glucose from salicin, resulting in no net endogenous loss of glucose from salicin (McLean et al. 2001; Pass et al. 2000), which may offset the GA cost of pinene. Salicin and pinene were part of the 4-way mixture, which also resulted in low GA excretion. Similar to our results, Marsh et al. (2006b) found that brushtail possums had greater DMI of a mixture of salicin and a monoterpene (cineole or *p-*cymene) than when offered a diet with two monoterpenes or individual monoterpenes or phenolics. Marsh et al. (2006b) might have found more consistent support for the detoxification limitation hypotheses because they used higher concentrations of PSMs for their mixture which might have put greater burden on detoxification pathways.

Our finding that deer did not eat more of the 4-way mixture than any of the 2-way mixtures, may reflect the complexity of detoxification that is oversimplified by the detoxification limitation hypothesis. For example, the two phases of physiological detoxification are not completely independent. Phase I reactions are often followed by conjugation from the Phase II reactions (Marsh et al. 2006a; Zhao et al. 2021). In addition, some PSMs require additional biotransformation beyond either Phase I and Phase II reactions, and these phases do not have to happen sequentially (Esteves et al. 2021; Phang-Lyn and Llerena 2025). GI microbes can also contribute to Phase I and II pathways, thus altering absorption by the host or by providing alternative source of glucuronic acid for conjugation (Marsh et al. 2006a; McLean and Duncan 2006). An alternative explanation for reduced consumption of the most diverse PSM diet is the phytochemical complementarity hypothesis (Rogosic et al. 2007) which postulates that plants should create a wide diversity of PSMs to increase the likelihood that they will produce the compound or mixture of compounds that will adequately deter a specific herbivore and provide defenses for a wider variety of herbivores. Given the potential for each PSM to inhibit CYPS and for quercetin to also activate CYPs, the 4-way mixture could create additive or synergistic toxic effects that herbivores avoid (Marsh et al. 2006b; McLean and Duncan 2006; Moore et al. 2015; Richards et al. 2016).

Overall, our findings have increased understanding of how mammalian herbivores respond to mixtures of PSMs, specifically in two species not previously investigated. Further strides in testing the predictions of the detoxification limitation hypothesis, especially in wild ruminants, requires a better understanding of biotransformation pathways for a wider variety of PSMs and how pathways of hosts and GI microbes intersect. In particular, comparing behavioral, physiological, and microbial responses to dietary PSMs among foregut and hindgut fermenters would enhance our understanding of the respective roles of host physiology and microbial communities in PSM tolerance in herbivores. Experimental trials that directly measure circulating and excreted metabolites or transformations by GI microbiomes using purified chemicals in vivo can be particularly difficult in large mammalian herbivores such as ruminants that are difficult to handle in a captive setting. However, future studies on wild ruminants that consume natural diets with diverse PSMs offer a unique opportunity to identify the mechanisms that promote or impede microbial partnerships, and could leverage the growing number of studies that describe whole genomes (London et al. 2022; Hallas et al. 2026), or quantify microbial networks (Srinivasan et al. 2024), to assess detoxification pathways of the host and the microbiome. Although no wild ruminant is considered an obligatory dietary specialist (Shipley et al. 2009), guilds of ruminant species (e.g., pronghorn [*Antilocapra Americana*], mule deer, and Rocky Mountain elk [*Cervus canadensis*] in sagebrush [*Artemisia tridentata*] ecosystems (Welch and Pederson 1981; Wambolt 2001) and populations within species (e.g., moose, Shipley 2010) are spread along the specialist-generalist continuum that would allow comparisons linking diet to microbes along a gradient of PSM exposure. Because concentrations and types of PSMs are expected to respond rapidly in the face of accelerating climate change (Moore et al. 2015; Verma and Shukla 2015), understanding how these changes might influence wild and domestic herbivores and microbiomes is critical for future food sources and population management.

## Data Availability

All data and code available on the first author’s Github (https://github.com/MooseKate/PSMs_and_Deer).
